# Serological profiles in nursery piglets colonized with *Staphylococcus aureus*

**DOI:** 10.1186/1297-9716-44-4

**Published:** 2013-01-22

**Authors:** Florence Crombé, Wannes Vanderhaeghen, Corné P de Vogel, Willem J Van Wamel, Kurt Barbé, Katleen Hermans, Freddy Haesebrouck, Patrick Butaye

**Affiliations:** 1 Veterinary and Agrochemical Research Centre (VAR), Department of Bacterial Diseases, Groeselenberg 99, Ukkel, Belgium; 2 Ghent University, Faculty of Veterinary Medicine, Department of Pathology, Bacteriology and Avian Diseases, Salisburylaan 133, Merelbeke, 9820, Belgium; 3 Erasmus Medical Centre, Departments of Medical Microbiology and Infectious Diseases, ‘s-Gravendijkwal 230, Rotterdam, The Netherlands; 4 Vrije Universiteit Brussel, Faculty of Engeneering, Deptartment of fundamental electricity and instrumentation (ELEC), Pleinlaan 2, Brussels, Belgium

## Abstract

At present, the immune response of pigs in relation to *Staphylococcus aureus* carriage is poorly understood. This study was aimed at investigating the dynamics of the anti-staphylococcal humoral immune response in methicillin-susceptible *S. aureus* (MSSA)-positive piglets and at assessing the effect of the experimental introduction of a methicillin-resistant *S. aureus* (MRSA) Sequence Type (ST) 398 strain. Therefore, serum samples were collected at different times from 31 weaned piglets originating from four different sows. Twenty-four out of the 31 piglets were challenged with MRSA ST398. The serum samples were analyzed for IgG antibodies to 39 *S. aureus* antigens, using a multiplex bead-based assay (xMAP technology, Luminex Corporation). Though antibody responses showed broad inter-individual variability, serological results appeared to be clustered by litter of origin. For most antigens, an age-related response was observed with an apparent increase in antibody titers directed against staphylococcal microbial surface components recognizing adhesive matrix molecules (MSCRAMM), which have been shown to play a role in *S. aureus* colonization. In most animals, antibody titers directed against staphylococcal toxins or immune-modulating proteins decreased with age, possibly reflecting the absence of bacterial invasion. The introduction of MRSA ST398 did not elicit a significant humoral immune reaction.

This study describes, for the first time, the humoral immune response in weaned pigs colonized with *S. aureus*.

## Introduction

*Staphylococcus aureus* is an opportunistic pathogen residing on the skin and mucous membranes of humans and several animal species [1,2]. In humans, it has been shown that nasal *S. aureus* carriage increases the risk of infection by a factor three, with patients being mostly affected by their autologous strain [3]. Conversely, a lower risk for bacteremia-related death has been reported in *S. aureus* carriers compared to non-carriers [3].

The differences in risk and outcome of *S. aureus* infections between human carriers and non-carriers have been associated with the humoral immune response, protecting carriers from bacteremia mediated death and non-carriers from *S. aureus* colonization. This response might include antibodies against staphylococcal microbial surface components recognizing adhesive matrix molecules (MSCRAMM) and against virulence factors involved in evading or destroying host defenses [4-8].

The extensive spread of livestock-associated methicillin-resistant *S. aureus* (LA-MRSA) clonal complex (CC) 398 and CC9 among pigs has raised questions on the mechanisms establishing the interactions between MRSA and pigs [9-11]. As in humans, the pigs’ immune system might play an important regulatory role. However, at present, knowledge on the (humoral) immune response of pigs to *S. aureus* is lacking. Therefore, our aim was to increase the understanding of this topic.

In the present study, we examined the kinetics of anti-staphylococcal antibodies in weaned piglets colonized with *S. aureus* by determining the levels of IgG antibodies to 9 MSCRAMM, 23 staphylococcal toxins and 7 immune-modulating proteins in serum samples. In addition, we investigated the effect of the introduction of a MRSA Sequence Type (ST) 398 strain on the piglets’ humoral immune response.

## Materials and methods

### Animals and serum samples

Venous blood samples were obtained from 31 healthy weaned cross-bred piglets (Landrace × Piétrain, Malèves-St. Marie, Belgium) previously described in a transmission study with MRSA strain C26 (ST398, *spa* type t011, SCC*mec* type V) [12]. Briefly, at 21 days of age, piglets obtained from four different sows (1–4), were transported to the experimental site and distributed into three experimental groups (1–3) consisting of eight piglets, and one negative control group of seven piglets. All three experimental groups included four male and female pairs originating from each of the four sows. The negative control group was composed of three male and female pairs, originating from sows 1–3, and a single female piglet originating from the fourth sow. The transmission study was performed by introducing two MRSA-positive animals (i.e. seeders that were inoculated with ~9 × 10^8^ CFU of MRSA strain C26 two days before their introduction), at 30 days of age, in each experimental group composed of six MRSA-negative piglets. Swab samples from all animals were collected separately from both anterior nares, the skin behind the ears and the perineum, during a period of six weeks to determine their *S. aureus* carriage state. For the present study, serum samples were taken before the start of the transmission experiment (six days before introduction of the MRSA positive animals – age: 24 days) and at 8, 15, 22, 29 and 36 days post introduction, corresponding to 38, 45, 52, 59 and 66 days of age.

Testing at 24 days of age revealed that all animals were positive for methicillin-susceptible *S. aureus* (MSSA) ST9 (*spa* type t3446 or t337). In all 24 animals that were included in the MRSA transmission study, MSSA and/or MRSA were detected until the end of the trial [12]. In the seven animals of the negative control group that were not colonized with MRSA strain C26, MSSA was not detected after the second sampling occasion, except once for one animal at the end [12]. To evaluate the effect of MRSA strain C26 introduction on the piglets’ humoral response, animals were classified as persistent MRSA carriers (*n* = 8) if ≥ 80% of the samples taken at different time points after the introduction of MRSA were MRSA positive. Animals were classified as non-MRSA carriers (*n* = 7) if all samples were MRSA negative; the other animals were classified as intermittent MRSA carriers (*n* = 16).

### *S. aureus* strain characteristics

The presence of 39 virulence-associated genes (Table 1) was determined in one MSSA isolate of each *spa* type originating from one randomly selected animal at 24 days of age and in MRSA strain C26 by DNA microarray by Alere Technologies GmbH, Jena, Germany [13-15].

**Table 1:** **DNA microarray results for the 39 *S. aureus *antigens studied by Luminex assay**

**Antigens of *S. aureus***	**Full name**	**DNA microarray results**		
		**ST398-V/t011**	**ST9/t3446**	**ST9/t337**
MSCRAMM				
ClfA	clumping factor A	+	+	+
ClfB	clumping factor B	+	+	+
FnbpA	fibronectin binding protein A	+	+	+
FnbpB	fibronectin binding protein B	+	+	+
IsdA	iron-responsive surface determinant A	+	+	+
IsdH	iron-responsive surface determinant H	ND	ND	ND
SasG	surface protein G	-	-	-
SdrD	serine-aspartate dipeptide repeat D	+	+	+
SdrE	serine-aspartate dipeptide repeat E	ND	ND	ND
Immune-modulating proteins				
CHIPS	chemotaxis inhibitory protein of *S. aureus*	-	-	-
SCIN	staphylococcal complement inhibitor	-	-	-
SSL1	*S. aureus* surface protein like 1	+	+	+
SSL3	*S. aureus* surface protein like 3	-*	+	+
SSL5	*S. aureus* surface protein like 5	+	+	+
SSL9	*S. aureus* surface protein like 9	+	+	+
SSL11	*S. aureus* surface protein like 11	-	-	-
Leucotoxins				
HlgB	haemolysin beta	+**	+	+
LukD	leukocidin D	-	-	-
LukE	leukocidin E	-	-	-
LukF	leukocidin F	+	+	+
LukS	leukocidin S	+	+	+
Staphylococcal enterotoxins				
α-toxin	alfa-toxin	+	+	+
SEA	staphylococcal enterotoxin A	-	-	-
SEB	staphylococcal enterotoxin B	-	-	-
SEC	staphylococcal enterotoxin C	-	-	-
SED	staphylococcal enterotoxin D	-	-	-
SEE	staphylococcal enterotoxin E	-	-	-
SEG	staphylococcal enterotoxin G	-	+	+
SEH	staphylococcal enterotoxin H	-	-	-
SEI	staphylococcal enterotoxin I	-	+	+
SEJ	staphylococcal enterotoxin J	-	-	-
SEM	staphylococcal enterotoxin M	-	+	+
SEN	staphylococcal enterotoxin N	-	+	+
SEO	staphylococcal enterotoxin O	-	+	+
SEQ	staphylococcal enterotoxin Q	-	-	-
SER	staphylococcal enterotoxin R	-	-	-
TSST-1	toxic shock syndrome toxin-1	-	-	-
Exfoliative toxins				
ETA	exfoliative toxin A	-	-	-
ETB	exfoliative toxin B	-	-	-

ND: Not determined.

*- for Staphylococcal Superantigen like protein 8/SSL3, + for Staphylococcal Superantigen like protein B3.

**+ for un-truncated Haemolysin Beta.

### Anti-staphylococcal antibody assay

Levels of IgG directed against 39 *S. aureus* proteins were quantified simultaneously using a bead-based flow cytometry technique (xMap; Luminex Corporation, Austin, TX, USA) based on a previously described method [5,6]. Briefly, serum samples were diluted 1:50 in PBS-BN (PBS, 1% bovine serum albumin, and 0.05% sodium azide [pH7.4]) for measurements of antigen-specific IgG. Per diluted sample, 50 μL was incubated with the different fluorescence-coloured antigen-coupled microspheres, i.e. 3000 beads per antigen per well, in a 96-well filter microplate (Millipore, Amsterdam, The Netherlands) for 35 min at room temperature on a Thermomixer plate shaker (Eppendorf, Nijmegen, The Netherlands). After washing the plate twice with PBS-BN and aspiration by vacuum manifold, 50 μL of a 1:100 dilution of R-phycoerythrin (RPE)-labelled anti-swine IgG (Jackson Immuno Research, Westgrove, PA, USA) was added. Following a second incubation (35 min at room temperature) and washing step, the microspheres were resuspended in 100 μL PBS-BN. Measurements were performed on the Luminex 100 instrument (BMD, Croissy-Beaubourg, France) using Luminex software (version 2.2). Tests were performed in independent duplicates and the median fluorescence intensity (MFI) values, reflecting semi-quantitative antibody levels, were averaged. Duplicates with a coefficient of variance (CV) higher than 25% were considered as not reproducible and deleted from the data set. If antibody binding was observed in the serum samples incubated with the control beads (i.e. beads without protein coupled on their surface), the nonspecific MFI values were subtracted from the antigen-specific results. Swine pooled serum, collected from 85 healthy pigs, incubated with protein-coupled beads was used as a positive control. Similarly, PBS-BN was included as a negative control.

### Statistical analysis

Independent-sample median test was performed to determine the distribution across the animals grouped by litter of origin with a 0.05 threshold at 24 days of age (SPPS software, version 19) [16].

The kinetics of anti-staphylococcal antibodies were analyzed using linear regression during a 6-week period. A linear mixed model including fixed and random effects was used by means of the MIXED procedure of SPPS software, version 19. The model was fitted using the Maximal Likelihood estimation (ML) method and used to evaluate the effect of MRSA ST398 introduction on the antibody profiles of the piglets. Age (in days), group (non-MRSA carriers vs. persistent and intermittent MRSA carriers) and gender (male vs. female) were introduced as fixed effects. To increase the degrees of freedom in the statistical analysis, the factor “age” was not included as a repeated measure, though the different sampling occasions are dependent, because of the limited data of the study. However, the interaction term “age*group” was added to correct for the time effect. In this way changes between groups over time could be observed. The piglets and their respective mothers (i.e. the interaction term “pig*sow”) were introduced as random effect to account for piglet and sow individual effects and disturbances.

## Results

### *S. aureus* strain characteristics

The presence of genes encoding virulence factors included in the antibody assay in the studied *S. aureus* strains is mentioned in Table 1.

### Anti-staphylococcal antibody response

#### Overall antibody response over time

IgG antibodies were detected in the positive control sample (swine pooled serum) for all but one (i.e. SEB) antigen. The negative control (PBS-BN) incubated with protein-coupled beads resulted in low MFI values (< 9). Serum incubated with control beads resulted in median MFI values of 208 (range: 83–606). Two samples were deleted from the data set, corresponding to serum of an animal at 24 days of age and one at 59 days of age, because the first one had high control bead levels (MFI-value of 1969) and the other one showed MFI-values similar to the PBS-BN. Both piglets belong to the persistent MRSA carrier group.

Median MFI values reflecting IgG levels against six out of 39 *S. aureus* antigens were nearly absent in the piglets’ serum samples, i.e. SEB, SEE, SEH, SEI, SEN and SEO (median MFI < 1) (data not shown). As a result, the antibody response against these antigens will not be further discussed.

**Figure 1:**
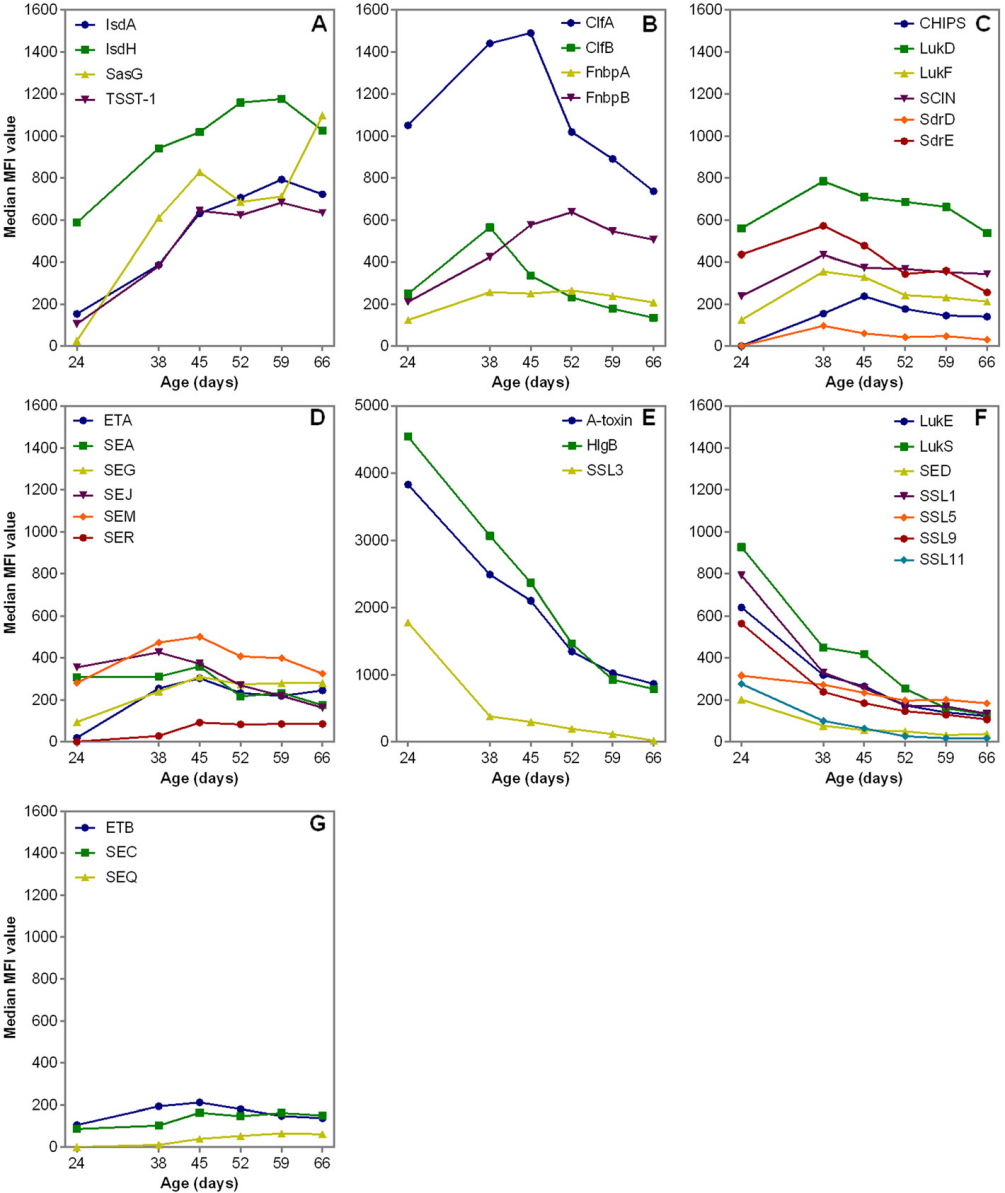
**Median of the median fluorescence intensity (MFI) values reflecting IgG levels for 33 *S. aureus *antigens in 31 piglets over 6 weeks.** Four trends were observed: increasing antibody levels (**A**), increasing antibody levels followed by a slow decline (**B,C,D**), decreasing antibody levels (**E,F**) and nearly no varying antibody levels over time (**G**).

As shown in Figure 1, four antibody response trends could be observed over the 6-week period: i. continuously increasing antibody levels over time (A), ii. increasing antibody levels followed by a slow decline over time (B,C,D), iii. a high antibody level at the start that decreased over time (E,F) and iv. nearly no varying antibody levels over time (G). In line with these observations, a significant “time” effect was observed (*p* < 0.05) for all but two of the 33 antigens (i.e. ETB and SEC) (data not shown).

#### Antigen-specific antibody levels classified per sow

**Figure 2:**
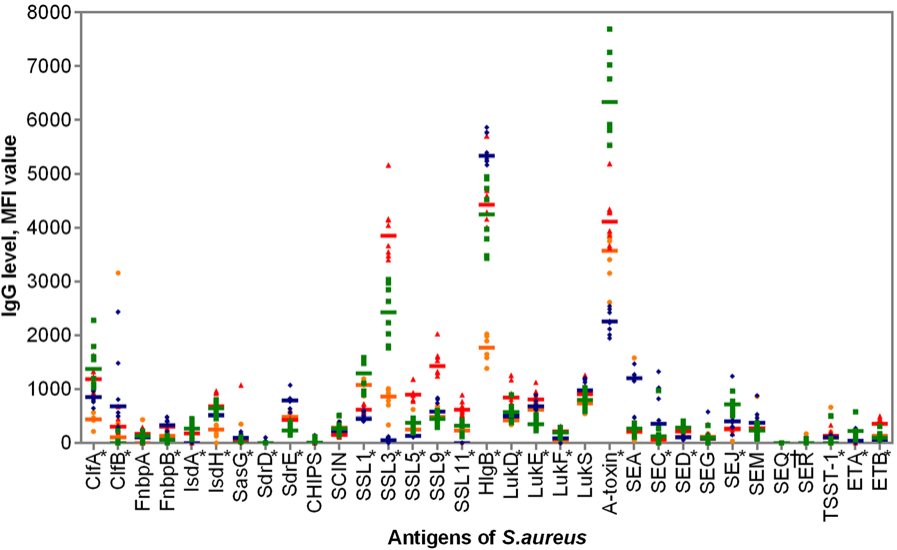
**Median fluorescence intensity (MFI) value reflecting IgG levels for 33 *S. aureus *antigens in 30 piglets classified per litter at 24 days of age.** Each dot represents a single piglet, green squares represent litter 1, blue diamonds represent litter 2, red triangles represent litter 3 and orange spheres represent litter 4. Median IgG levels are indicated by horizontal lines. *Significant with independent-sample median test across animals grouped by litter at 24 days of age (α-toxin, FnbpB, SED, SSL1, SSL3 and SSL5, *p* < 0.001; ClfA, ClfB, ETA, HlgB, IsdA, IsdH, LukE, LukF, SdrE, SEJ, SSL9 and SSL11, *p* < 0.005; ETB, LukD, *p* < 0.01; SasG, SdrD, SEC and TSST-1, *p* < 0.05); † Unable to compute for SEQ.

**Figure 3:**
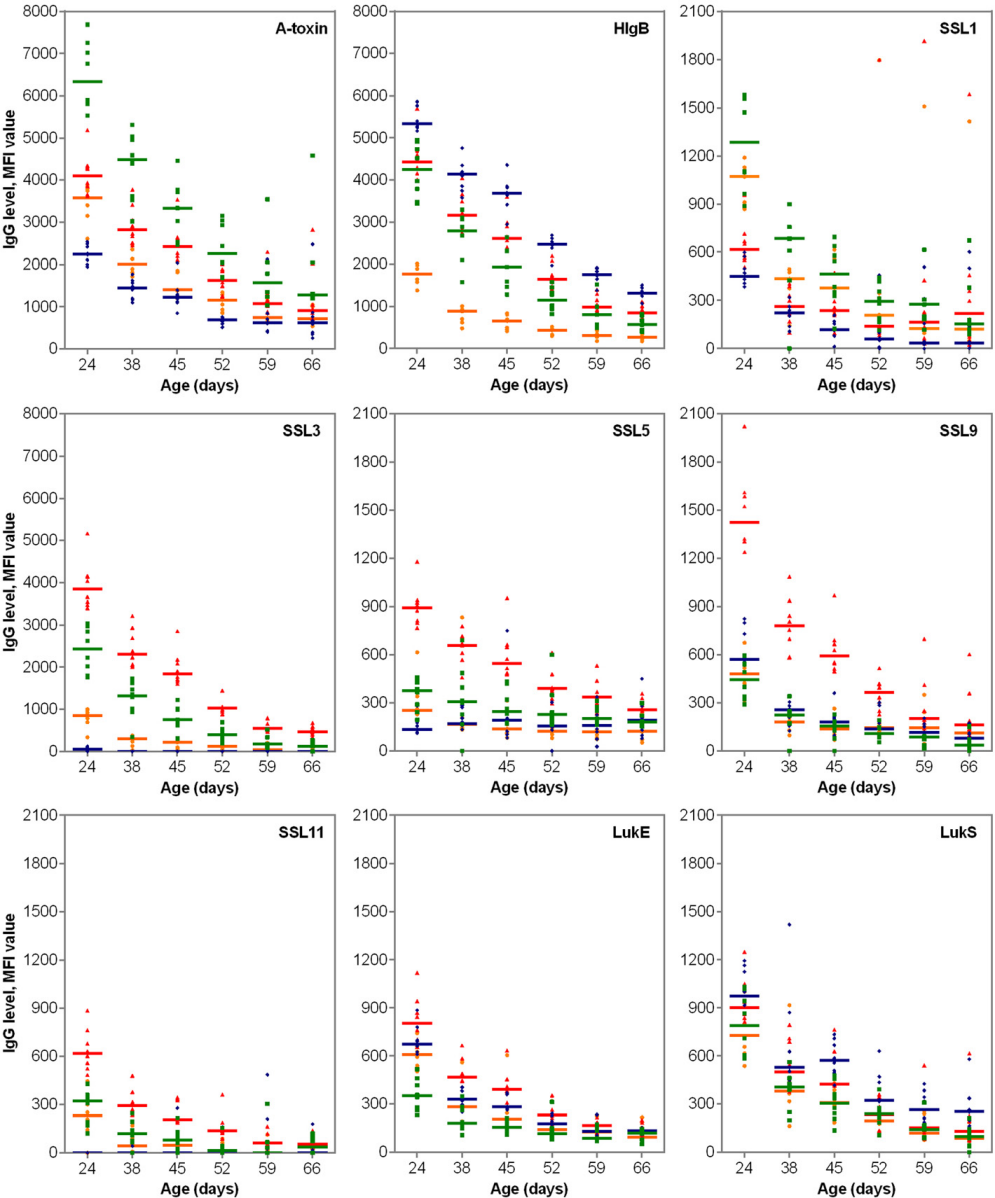
**Median fluorescence intensity (MFI) values reflecting IgG levels against α-toxin, HlgB, SSL1, SSL3, SSL5, SSL9, SSL11, LukE and LukS in 31 piglets classified per litter over a 6-week period.** Each dot represents a single piglet, green squares represent litter 1, blue diamonds represent litter 2, red triangles represent litter 3 and orange spheres represent litter 4. Median IgG levels are indicated by horizontal lines.

**Figure 4:**
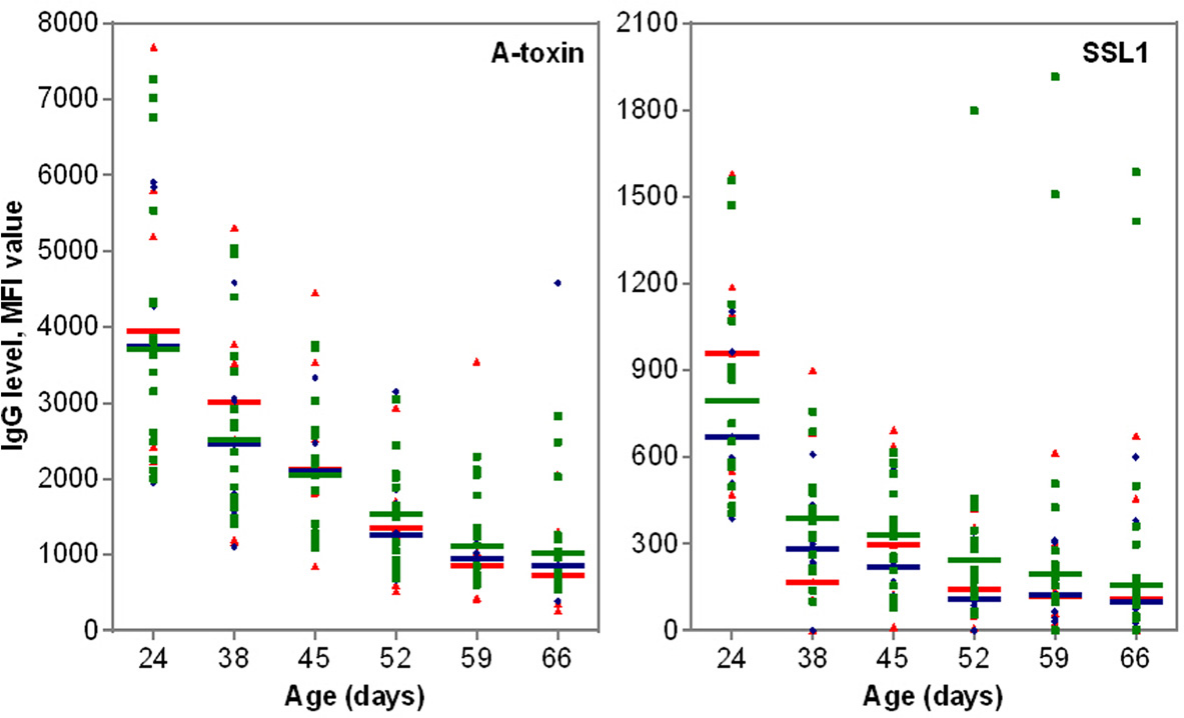
**Median fluorescence intensity (MFI) values reflecting IgG levels against α-toxin and SSL1 in 31 piglets classified per MRSA carrier group over a 6-week period.** Each dot represents a single piglet, green squares represent intermittent MRSA carriers (*n* = 16); blue diamonds represent persistent MRSA carriers (*n* = 8), red triangles represent non-MRSA carriers (*n* = 7). Median IgG levels are indicated by horizontal lines.

Although antibody levels varied among the animals, the IgG levels directed against several antigens appeared to be grouped by litter of origin (Figure 2). These data are presented in an additional data file (see Additional file 1). An independent-sample median test revealed that the distributions across the animals grouped by litter of origin were significantly different for 24 out of the 33 antigens tested at 24 days of age (*p* < 0.05) (Figure 2). Also, when focusing on the antigens with decreasing global antibody profiles (i.e., α-toxin, HlgB, SSL1, SSL3, SSL5, SSL9, SSL11, LukE and LukS), a litter-related decrease was perceived over time (Figure 3). Remarkably though as a general trend, the levels of antibodies against α-toxin and SSL1 declined, it appeared that IgG levels increased for certain animals at 59 and 66 days of age (Figure 4).

#### Effect of MRSA introduction on antibody profiles

The ‘group’ effect was significant for one antigen (i.e. ClfB, *p* = 0.014), with mean IgG levels being significantly lower in persistent MRSA carriers compared with non-MRSA carriers (*p* = 0.031). Mean IgG levels directed against ClfB were lower (though not significant) in intermittent MRSA carriers compared with non-MRSA carriers (*p* = 0.09) (data not shown).

**Figure 5:**
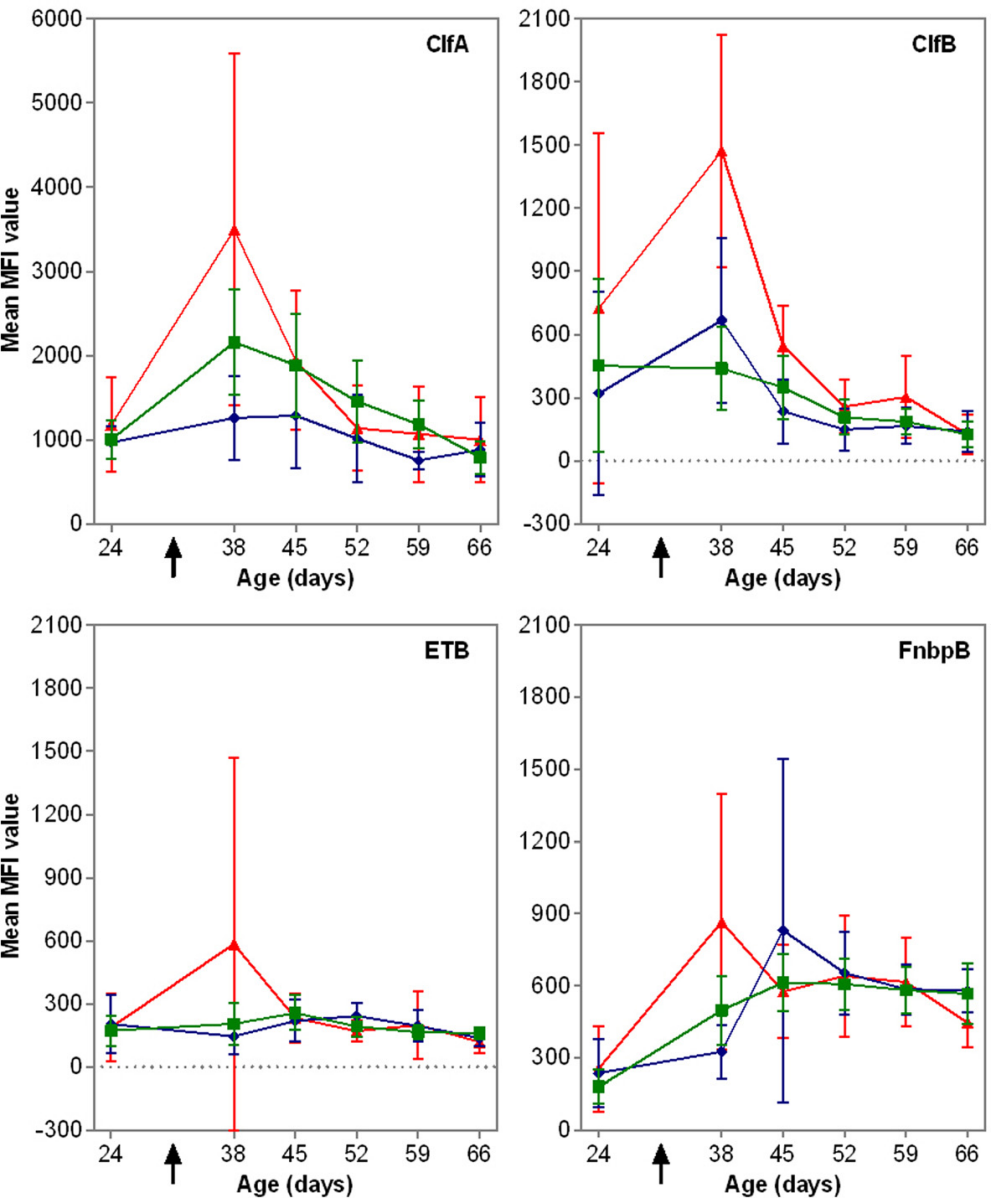
**Mean of the median fluorescence intensity (MFI) values reflecting IgG levels for ClfA, ClfB, ETB and FnbpB per MRSA carrier group over time.** Green squares represent intermittent MRSA carriers (*n* = 16); blue diamonds represent persistent MRSA carriers (*n* = 8), red triangles represent non-MRSA carriers (*n* = 7). Data are presented as mean±95% CI. ↑: MRSA introduction.

The humoral immune response to ClfA, ClfB, ETB and FnbpB differed significantly between groups over time (age*group interaction; *p* < 0.001, *p* = 0.001, *p* = 0.035 and *p* = 0.006, respectively). At the peak of the IgG response (38 days of age), antibody levels directed against ClfA and ETB were significantly higher in non-MRSA carriers compared to persistent MRSA carriers (*p* < 0.001; *p* = 0.003) and intermittent MRSA carriers (*p* = 0.007; *p* = 0.006) (Figure 5). For ClfB, antibody levels were significantly higher in non-MRSA carriers compared to intermittent MRSA carriers (*p* < 0.001) though not compared to persistent MRSA carriers (*p* = 0.12) (Figure 5). Conversely, for FnbpB, antibody levels were significantly higher in non-MRSA carriers compared to persistent MRSA carriers (*p* = 0.003) though not compared to IC (*p* = 0.058) (Figure 5). However, for ETB, one non-MRSA carrier had a very high IgG level at 38 days of age and when deleting this record from the dataset a significant difference was no longer observed (age*group interaction; *p* = 0.77).

When deleting an additional non-MRSA carrier from the dataset, with high levels directed against ClfA and FnbpB at 38 days of age, a significant difference was also no longer observed (age*group interaction; *p* = 0.094 and *p* = 0.075).

For each sampling occasion, mean IgG levels ± 95% CI of intermittent MRSA carriers, persistent MRSA carriers and non-MRSA carriers are presented in an additional data file (see Additional file 2).

## Discussion

To our knowledge, this is the first study on humoral immune response against *S. aureus* in weaned pigs. We used a multiplex bead-based assay (xMAP technology, Luminex Corporation) to simultaneously quantify antibodies against 39 staphylococcal proteins in small-volume serum samples. This high-throughput immunological test is of particular interest since it gives the opportunity to simultaneously study the humoral response of healthy or diseased individuals to a specific pathogen towards large sets of antigens [5,6,17,18].

Antibodies against all but one of the 39 antigens (i.e. SEB) were detected in the swine pooled serum standard positive control. Therefore, the present study does not allow drawing conclusions with regards to serological responses to SEB. However, given that SEB is generally absent in *S. aureus* strains of animal origin [19], which was also the case for the investigated strains, it is unlikely that pigs produced antibodies against this antigen.

Four trends were observed among the dynamics of anti-staphylococcal antibody response in all piglets colonized with *S. aureus*: i. a main increase in response over time, ii. an increase in response followed by a slow decline, iii. a main decrease in response over time or iv. almost no change in response. Given that the observational period of the present study started at weaning, it is likely that serum IgG directed against certain antigens are de novo synthesized by the piglets whereas others are of maternal origin [20,21]. Increasing antibody titers indicate de novo synthesis, whereas antibodies with decreasing titers are most probably from maternal origin. Interestingly, most antibodies with increasing levels were directed to antigens belonging to the family of the MSCRAMM, which are known to be expressed by actively dividing bacteria and are essential in the colonization process of *S. aureus*[2,22-24]. This indicates that maternal antibodies do not protect piglets from colonization. Indeed, it has been described that piglets derived from *S. aureus*-positive sows are more likely to become *S. aureus* colonized [25]. In contrast, antibodies with decreasing levels were mostly directed against staphylococcal toxins or immune-modulating proteins interfering with host defences and playing a role during staphylococcal invasion [5,6].

For certain antigens, antibody response was observed though the corresponding genes were not detected on the microarray in the investigated *S. aureus* strains. Hence, it must be noted that the microarray technology used here bears some limitation as it only detects genes matching the probes printed on the array. Given that this composite multi-strain array covers allelic variants of genes of *S. aureus* genomes of diverse human and bovine lineages, allelic variants specific for porcine ST398 and ST9 strains might be missing [14,15,26]. For example, Schijffelen [27] reported that a *scn* gene homologue encoding SCIN was present on a novel ‘animal-specific’ staphylococcal pathogenicity island (SaPI-S0385), in MRSA ST398 strain S0385 that was not included on the array. Similarly, a *chp* gene homologue coding for Fprl1 inhibitory protein (Flipr) [28], a CHIPS homologue, has been described in MRSA ST398 strain S0385. Another explanation might be that other non-*S. aureus* staphylococcal species, present on the piglets’ mucosae, express *S. aureus* like antigens. Several studies reported a high occurrence of non-*S. aureus* staphylococci in the nares of pigs [29-32] and it has been shown that staphylococcal species share a common reservoir of virulence factors [33-35]. Notably, *S. aureus* and *Staphylococcus hyicus*, a pathogen causing exudative epidermitis in pigs and a staphylococcal species commonly found in pigs’ endogenous flora [29,36], carry related ETB [33], which might explain the high IgG levels observed in a 38-day-old animal in the present study though ETB was absent in the investigated *S. aureus* strains.

On the contrary, IgG levels against several enterotoxins (i.e. SEI, SEO, SEN) – encoded on the enterotoxin gene cluster (*egc*) – were nearly absent though genes coding for these factors were present in the investigated *S. aureus* strains. Staphylococcal enterotoxins are likely to be mainly expressed during infection and less during colonization, which might explain the absence of antibodies in sera from healthy pigs. Antibodies against *egc* enterotoxins are also rarely detected in sera from healthy humans [37].

The introduction of MRSA strain C26 at weaning appeared not to increase the anti-staphylococcal antibody titers. These observations indicate a low immunogenic potential of strain C26 during colonization. However, since the observational period is at weaning, maternal antibodies might have interfered with the piglets’ response [20,21]. Furthermore, conventional pigs naturally colonized with *S. aureus* and having normal microbiota, including various staphylococcal species [30,38], were used in the present study. Though our trial resembles MRSA colonization as noticed in the field situation on many pig herds [9,25], using such pigs complicates the interpretation of the immune response against the MRSA ST398 strain used here. Indeed, in the present study, IgG levels directed against ClfA, ClfB and FnbpB were significantly lower in persistent MRSA carriers and/or intermittent MRSA carriers compared to non-MRSA carriers at 38 days of age. This might indicate that MRSA ST398 strain C26 suppresses systemic antibody responses against certain *S. aureus* antigens thus possibly facilitating its colonization. However, since extensive inter-individual differences were observed, these significant effects appeared to result from few animals for ClfA and FnbpB. Future studies with caesarean-derived colostrum-deprived piglets may help to further elucidate the immune response associated with MRSA colonization, although this is a more artificial model [39]. In addition, the immunogenic potential of highly virulent MRSA clones such as the community-associated MRSA-like ST8 clone, known as USA300, recently isolated from pigs in Peru [40] and USA [41], are interesting issues to consider in further studies. In that way, the effect of strongly immunogenic extracellular toxins such as Panton-Valentine leukocidin, a cytotoxin which is probably associated with necrotizing lesion development, and additional enterotoxins on the humoral immune response of piglets could be studied.

Since the focus has been set on serum IgG which dominates the systemic immunity, the possible role of IgA involved in the mucosal immune system, remains to be investigated [20,42-44]. Also, the cellular immune response after *S. aureus* colonization should be investigated.

In conclusion, this is the first report on the humoral immune response in weaned pigs colonized with *S. aureus*. Antibody titers directed against MSCRAMM increased over time whereas antibody titers directed against staphylococcal toxins or immune-modulating proteins decreased, which might partly reflect the role of specific antigens in *S. aureus* colonization. The introduction of MRSA ST398 did not elicit a significant humoral immune reaction. The presence of maternally derived immunity and other staphylococcal species in the piglets’ microbiota may have influenced the results of the present study.

## Competing interests

The authors declare that they have no competing interests.

## Authors’ contributions

Experimental design and planning: FC, KH, FH, PB; animal experiments: FC; serological analysis CPDV, WJVW; data processing and statistical analysis FC, CPDV, WJVW, KB; drafting of the manuscript: FC, WV; critical revision of the manuscript: CPDV, WJVW, KB, KH, FH, PB. All authors read and approved the manuscript.

## Supplementary Material

Additional file 1 Median fluorescent intensity (MFI) values reflecting antigen-specific immunoglobulin (Ig) G levels between litters at 24 days of age. Click here for file

Additional file 2 Median fluorescent intensity (MFI) values reflecting antigen-specific immunoglobulin (Ig) G levels between intermittent MRSA carriers, persistent MRSA carriers and non-MRSA carriers during 6 weeks. Click here for file
