# Supplementary material for: Serological profiles in nursery piglets colonized with Staphylococcus aureus
Source: Vet Res. 2013 Jan 22;44(1):4. doi: 10.1186/1297-9716-44-4 (PMC3558462; doi:10.1186/1297-9716-44-4)
Supplement: Additional file 1 — Median fluorescent intensity (MFI) values reflecting antigen-specific immunoglobulin (Ig) G levels between litters at 24 days of age. [file 1297-9716-44-4-S1.doc]

**Additional file 1 Median fluorescent intensity (MFI) values reflecting antigen-specific immunoglobulin (Ig) G levels between litters at 24 days of age.**

| **Antigen** | **MFI value. median (range)** | | | | ***p*-value*** |
| --- | --- | --- | --- | --- | --- |
|  | **Litter 1** | **Litter 2** | **Litter 3** | **Litter 4** |  |
| ClfA | 1368 (1023.5-2270.25) | 850.38 (636.50-1086.50) | 1176 (918.00-1626.25) | 442 (215.25-902.25) | 0.002 |
| ClfB | 0 (0-232.5) | 677.75 (294.75-2428.5) | 301.88 (19-673.25) | 105 (0-3156) | 0.001 |
| FnbpA | 134.25 (10.25-222.75) | 108.5 (0-168.75) | 170.63 (0-293.5) | 0 (0-435.5) | NS |
| FnbpB | 63.13 (0-137.25) | 334.5 (45-476.25) | 303.5(278.5-439.75) | 130.5 (0-306.5) | <0.001 |
| IsdA | 266.38 (67-440.75) | 1.75 (0-184.5) | 179.13 (0-340.5) | 0 (0-396.25) | 0.003 |
| IsdH | 642.13 (482.75-793) | 512.13 (450.5-667.25) | 680.38 (611-962) | 246.13 (0-538.25) | 0.001 |
| SasG | 2.63 (0-106.5) | 96.75 (0-225) | 23.13 (0-1069.25) | 79.63 (0-352) | 0.029 |
| SdrD | 0 (0) | 0 (0-99.25) | 0 (0) | 0 (0) | 0.027 |
| SdrE | 230.13 (149.5-399.75) | 784.13 (563.75-1065.5) | 435.50 (306.25-580.5) | 474.13 (351.75-575.5) | 0.001 |
| CHIPS | 6.75 (0-116.75) | 9.38 (0-143.25) | 0.5 (0-97.50) | 0 (0-30.5) | NS |
| SCIN | 271.63 (137.5-507) | 228.25 (140-398.5) | 155 (108.25-291.5) | 282.5 (138-397) | NS |
| SSL1 | 1285.88 (886.5-1579.75) | 451.13 (386-599.25) | 617.13 (507.5-956.75) | 1070.75 (868.5-1189) | <0.001 |
| SSL3 | 2426.38 (1762.75-3036.00) | 51.88 (0-120) | 3854.5 (3404.75-5167) | 852 (335.75-1002.75) | <0.001 |
| SSL5 | 376.88 (194.75-458.25) | 134 (110.5-281.25) | 892.13 (767.25-1178.75) | 251.75 (188.5-617) | <0.001 |
| SSL9 | 446.13 (290.5-595) | 572.25 (536.25-825) | 1423.88 (1239.25-2022) | 479.75 (297.25-675) | 0.001 |
| SSL11 | 323.88 (121-428.5) | 0 (0) | 616.5 (483.5-885) | 230.5 (0-444.5) | 0.001 |
| HlgB | 4245.63 (3436.5-4950) | 5338.38 (5166.5-5861.25) | 4430.25 (4007-5698.25) | 1768.13 (1378.5-2018.25) | 0.002 |
| LukD | 572.13 (379-885) | 498.75 (390.75-607.5) | 842.88 (678.5-1250.75) | 418.13 (339.5-649.25) | 0.005 |
| LukE | 350.38 (232.25-460.5) | 675.75 (518.5-884.25) | 803.13 (656.5-1119.25) | 609.50 (504.25-742) | 0.001 |
| LukF | 192.5 (83.75-217.5) | 89.13 (0-216) | 217.88 (195-315.5) | 13 (0-79.75) | 0.001 |
| LukS | 787.88 (583.25-1030.25) | 973.5 (917.25-1194.25) | 901.25 (814.5-1251) | 728.25 (537.5-1001) | NS |
| α-toxin | 6335.5 (5534.5-7685.25) | 2248 (1945.75-2535.5) | 4109.25 (3622.75-5193.25) | 3576.25 (2611.25-3857.75) | <0.001 |
| SEA | 264.63 (110.75-383.5) | 1199.25 (220-1464) | 226.63 (125.25-371) | 203.75 (49.25-1574.75) | NS |
| SEC | 126.75 (0-968.5) | 356.13 (72.75-1320.25) | 71.13 (0-140.75) | 3.13 (0-283.5) | 0.022 |
| SED | 283.38 (211-411) | 102.00 (57-128.75) | 216.88 (197.25-319.5) | 115.5 (80-145.5) | <0.001 |
| SEG | 99.75 (0-325.5) | 88.13 (52-160.25) | 76.75 (10.5-346.5) | 117.63 (22.5-176) | NS |
| SEJ | 710 (509-957.25) | 406.75 (150-1238.25) | 268.25 (172-324.5) | 252.25 (35.25-674.75) | 0.001 |
| SEM | 234.63 (77.25-372.25) | 374.75 (229.25-873.5) | 268.63 (150.5-338.25) | 275.88 (128.5-861.5) | NS |
| SEQ | 0 (0) | 0 (0) | 0 (0) | 0 (0) | † |
| SER | 0 (0-89) | 0.63 (0-68.25) | 0 (0-89) | 0 (0-170) | NS |
| TSST-1 | 3.38 (0-496.25) | 112.25 (14.25-148.25) | 130.38 (94-332) | 100.38 (0-656) | 0.029 |
| ETA | 218.38 (57.75-572.5) | 42.75 (0-273.75) | 0 (0) | 5 (0-123.5) | 0.001 |
| ETB | 95.25 (55.25-308.5) | 62 (41-117) | 363.63 (307.5-491.75) | 132.13 (74.5-316.5) | 0.005 |

*Difference in antigen-specific MFI-values between litters were considered to be statistically significant at *p* < 0.05 (Independent-sample median test).

NS: not statistically significant; †: Unable to compute for SEQ.
