# Supplementary material for: Serological profiles in nursery piglets colonized with Staphylococcus aureus
Source: Vet Res. 2013 Jan 22;44(1):4. doi: 10.1186/1297-9716-44-4 (PMC3558462; doi:10.1186/1297-9716-44-4)
Supplement: Additional file 2 — Median fluorescent intensity (MFI) values reflecting antigen-specific immunoglobulin (Ig) G levels between intermittent MRSA carriers, persistent MRSA carriers and non-MRSA carriers during 6 weeks. [file 1297-9716-44-4-S2.doc]

**Additional file 2** Median fluorescent intensity (MFI) values reflecting antigen-specific immunoglobulin (Ig) G levels between intermittent MRSA carriers, persistent MRSA carriers and non-MRSA carriers during 6 weeks.

| **Antigen** | **Age (days)** | **MFI value, mean (95% CI)** | | |  |
| --- | --- | --- | --- | --- | --- |
|  |  | **Intermittent MRSA carriers** | **Persistent MRSA carriers** | **Non-MRSA carriers** | |
| ClfA | 24 | 1005.7 (770.17-1241.24) | 969.79 (781.35-1158.22) | 1181.64 (617.611-1745.68) | |
|  | 38 | 2157.2 (1534.34 - 2780.07) | 1262.66 (764.64-1760.67) | 3498.42 (1415.83-5581) | |
|  | 45 | 1888.63 (1283.15-2494.12) | 1291.19 (671.64-1910.73) | 1942.18 (1116.69-2767.67) | |
|  | 52 | 1458.68 (971.83-1945.54) | 1017.56 (495.79-1539.33) | 1141.86 (637.5-1646.22) | |
|  | 59 | 1186.37 (905.08-1467.65) | 758.71 (656.55-860.87) | 1072.43 (504.34-1640.52) | |
|  | 66 | 794.97 (602.9-987.03) | 886 (566.47-1205.53) | 1004.71 (502.88-1506.55) | |
| ClfB | 24 | 453.59 (44.77-862.42) | 321.21 (-163.64-806.07) | 724.64 (-109.12-1558.4) | |
|  | 38 | 439.56 (243.15-635.98) | 667.19 (276.34-1058.04) | 1473.21 (920.24-2026.18) | |
|  | 45 | 350.06 (201.12-499.01) | 235.69 (84.57-386.8) | 544.93 (355.12-734.74) | |
|  | 52 | 207.41 (123.77-291.04) | 149.19 (47.6-250.78) | 258.25 (129.17-387.33) | |
|  | 59 | 186.22 (125.71-246.73) | 165.29 (79.51-251.07) | 304.71 (108.89-500.54) | |
|  | 66 | 125.72 (65.1-186.34) | 139.84 (41.69-24) | 126.32 (34.44-218.2) | |
| FnbpA | 24 | 133.39 (70.03-196.75) | 62.75 (-7.77-133.27) | 146.71 (57.91-235.52) | |
|  | 38 | 350.97 (228.64-473.3) | 182.97 (75.083-290.85) | 398.54 (171.11-625.96) | |
|  | 45 | 403.41 (245.8-561.02) | 176.81 (71.47-282.15) | 285.29 (178.52-392.05) | |
|  | 52 | 366.55 (244.39-488.71) | 228.09 (145.15-311.04) | 264.86 (167.37-362.35) | |
|  | 59 | 301.58 (216.8-386.36) | 225.96 (130.13-321.8) | 322.93 (80.67-565.19) | |
|  | 66 | 253.84 (186.22-321.47) | 209.63 (148.38-270.87) | 196.14 (100.75-291.53) | |
| FnbpB | 24 | 179.73 (109.51-249.96) | 236.68 (94.88-378.47) | 255.75 (78.75-432.75) | |
|  | 38 | 498.11 (354.36-641.86) | 325 (214.05-435.95) | 866.71 (334.71-1398.72) | |
|  | 45 | 613.77 (493.17-734.36) | 830.29 (116.19-1544.38) | 576.04 (383.34-768.73) | |
|  | 52 | 607.03 (500.6-713.47) | 652.94 (481.55-824.33) | 640.54 (389.08-891.99) | |
|  | 59 | 581.64 (482.73-680.56) | 585.5 (480.31-690.69) | 615.29 (429.4-801.17) | |
|  | 66 | 567.59 (441.34-693.85) | 579.53 (489.85-669.22) | 446.39 (343.87-548.92) | |
| IsdA | 24 | 169.41 (87.667-251.15) | 120.86 (-8.1481-249.86) | 125.89 (8-243.78) | |
|  | 38 | 429.83 (259.26-600.4) | 431.91 (266.38-597.43) | 502 (265.77- 738.23) | |
|  | 45 | 613.97 (449.68-778.26) | 760.16 (246.87-1273.44) | 571.11 (350.09-792.12) | |
|  | 52 | 737.95 (521.28-954.63) | 792.06 (433.5-1150.62) | 737.11 (478.32-995.89) | |
|  | 59 | 893.7 (687.29-1100.11) | 864.86 (566.74-1162.98) | 977.25 (429.23-1525.27) | |
|  | 66 | 878.25 (585.57-1170.93) | 754.07 (475.74-1032.4) | 906.93 (572-1241.86) | |
| IsdH | 24 | 526.36 (412.89-639.83) | 557.89 (384.2-731.59) | 647.82 (386.62-909.02) | |
|  | 38 | 1000.54 (859.91-1141.16) | 729.78 (573.3-886.26) | 1208.14 (749.37-1666.92) | |
|  | 45 | 1048.89 (893.31-1204.47) | 926.44 (597.99-1254.89) | 1291.11 (707.76-1874.46) | |
|  | 52 | 1114.88 (968.09-1261.66) | 1149.25 (895.02-1403.48) | 1174.93 (632.3-1717.56) | |
|  | 59 | 1231.29 (997.09-1465.48) | 1046.33 (888.38-1204.29) | 1506.96 (705.19-2308.74) | |
|  | 66 | 1178.81 (906.2-1451.43) | 1067.28 (743.95-1390.62) | 1357.54 (794.82-1920.26) | |
| SasG | 24 | 86.55 (33.01-140.08) | 86.5 (-34.23-207.23) | 190.71 (-175.41-556.84) | |
|  | 38 | 1034.09 (525.81-1542.38) | 392.16 (276.28-508.04) | 837.89 (151.93-1523.86) | |
|  | 45 | 976.84 (744.8-1208.89) | 796.75 (412.28-1181.22) | 925.5 (299.57-1551.43) | |
|  | 52 | 681.5 (524.52-838.48) | 894.53 (644.22-1144.85) | 666.54 (431.47-901.6) | |
|  | 59 | 672.28 (505.81-838.75) | 872.57 (640.93-1104.21) | 923.36 (571.62-1275.09) | |
|  | 66 | 1217.89 (913.15-1522.64) | 1337.03 (777.53-1896.53) | 1551.68 (794.45-2308.91) | |
| SdrD | 24 | 6.52 (-6.68-19.71) | 5.71 (-8.27-19.7) | 0 (0) | |
|  | 38 | 86.41 (10.58-162.23) | 220.28 (38.68-401.89) | 196.61 (35.92-357.3) | |
|  | 45 | 66.09 (20.6-111.59) | 102.69 (-9.43-214.8) | 86.61 (32.06-141.15) | |
|  | 52 | 72.31 (6.27-138.36) | 123.44 (8.9-237.97) | 69.29 (3.72-134.85) | |
|  | 59 | 62.84 (25.23-100.45) | 94.32 (20.61-168.04) | 118.68 (-14.2-251.56) | |
|  | 66 | 62.34 (25.53-99.16) | 52.41 (8.1-96.72) | 20.82 (-2.07-43.71) | |
| SdrE | 24 | 489.84 (363.27-616.42) | 387.75 (188.71-586.79) | 537.71 (333.66-741.77) | |
|  | 38 | 646.97 (499.14-794.8) | 478.5 (419.21-537.79) | 793.39 (488.77-1098.01) | |
|  | 45 | 472.38 (378.58-566.17) | 396.25 (311.28-481.22) | 599.93 (368.6-831.25) | |
|  | 52 | 423.95 (282.41-565.5) | 332.16 (283.19-381.12) | 453.29 (318.71-587.86) | |
|  | 59 | 386.45 (298.23-474.68) | 356.96 (260.17-453.76) | 500.82 (182.61-819.03) | |
|  | 66 | 343.98 (185.45-502.52) | 284.78 (208.97-360.59) | 357.39 (227.49-487.29) | |
| CHIPS | 24 | 15.95 (2.56-29.34) | 19.25 (-20.67-59.17) | 25.25 (-24.15-74.65) | |
|  | 38 | 224 (151.58-296.42) | 135.81 (15.41-256.21) | 165.43 (110.08-220.78) | |
|  | 45 | 253.42 (198.84-308.01) | 200.69 (76.3-325.08) | 238.68 (105.22-372.14) | |
|  | 52 | 196.89 (139.16-254.62) | 160.16 (82.17-238.14) | 208.39 (74.90-341.88) | |
|  | 59 | 174.25 (122.06-226.44) | 148.18 (96.98-199.38) | 238.82 (94.657-382.99) | |
|  | 66 | 122.58 (86.62-158.54) | 131.38 (96.06-166.69) | 216.93 (133.61-300.25) | |
| SCIN | 24 | 258.73 (195.94-321.53) | 194.43 (134.89-253.97) | 245.07 (182.51-307.64) | |
|  | 38 | 443.17 (352.79-533.55) | 459.11 (327.29-590.92) | 352.61 (185.15-520.06) | |
|  | 45 | 382.13 (319.17-445.08) | 423.25 (283.91-562.59) | 312.75 (232.87-392.63) | |
|  | 52 | 380.02 (305.58-454.45) | 373.97 (267.82-480.12) | 317.29 (187.67-446.9) | |
|  | 59 | 401.38 (337.96-464.81) | 302.61 (265.84-339.38) | 350.89 (228.13-473.65) | |
|  | 66 | 393.59 (328.51-458.68) | 322.37 (207.69-437.06) | 333.18 (230.31-436.05) | |
| SSL1 | 24 | 861.47 (658.4-1064.54) | 757.11 (491.68-1022.53) | 896.68 (498.48-1294.88) | |
|  | 38 | 398.03 (293.52-502.54) | 313.47 (161.86-465.08) | 341.21 (31.79-650.63) | |
|  | 45 | 333.83 (236.75-430.91) | 247.81 (126.26-369.37) | 335.79 (100.1-571.48) | |
|  | 52 | 436.41 (145.46-727.36) | 131.63 (55.26-207.99) | 187.96 (47.76-328.17) | |
|  | 59 | 383.94 (96.40-671.47) | 154.79 (43.15-266.42) | 179.43 (-20.23-379.09) | |
|  | 66 | 335.38 (83.43-587.33) | 182.34 (12.2-352.49) | 206.64 (-32.76-446.04) | |
| SSL3 | 24 | 1782.06 (945.17-2618.95) | 1799.79 (327.81-3271.76) | 2132.89 (353.35-3912.44) | |
|  | 38 | 1006.39 (469.87-1542.91) | 878.47 (33.92-1723.02) | 1148.5 (-4.36-2301.36) | |
|  | 45 | 726.33 (277.82-1174.85) | 641.03 (-22.36-1304.42) | 943.32 (-27.02-1913.66) | |
|  | 52 | 416.41 (167.98-664.83) | 341.53 (-3.3-686.36) | 480 (-18.11-978.11) | |
|  | 59 | 223.95 (90.83-357.07) | 205.79 (-42.44-454.01) | 284.82 (-18.65-588.3) | |
|  | 66 | 146.69 (38.41-254.97) | 143.06 (-34.27-320.39) | 195.5 (-51.89-442.89) | |
| SSL5 | 24 | 417.5216 (259.55-575.48) | 421 (142.28-699.73) | 498.61 (130.15-867.07) | |
|  | 38 | 375.16 (251.40-498.91) | 233.47 (65.61-401.32) | 434.71 (157.1-712.33) | |
|  | 45 | 374.66 (242.33-506.98) | 264.84 (126-403.69) | 283.07 (82.74-483.41) | |
|  | 52 | 245.58 (163.73-327.43) | 223.56 (122.51-324.62 | 289.61 (129.07-450.15) | |
|  | 59 | 225.13 (164.18-286.07) | 188.96 (102.06-275.87) | 249.14 (112.5-385.79) | |
|  | 66 | 206.61 (162.65-250.57) | 221.56 (121.67-321.46) | 170.64 (106.59-234.7) | |
| SSL9 | 24 | 735.44 (499.07-971.8) | 779.82 (348.92-1210.73) | 893.11 (357.51-1428.71) | |
|  | 38 | 344.3 (205.14-483.46) | 340.88 (92.97-588.78) | 370.96 (34.08-707.85) | |
|  | 45 | 266.3 (160.74-371.86) | 263.84 (54.08-473.6) | 329.39 (41.54-617.24) | |
|  | 52 | 179.98 (113.7-246.27) | 206.69 (115.09-298.29) | 208.93 (75.47-342.39) | |
|  | 59 | 163.97 (78.86-249.08) | 158.46 (62.61-254.32) | 160.43 (54.24-266.61) | |
|  | 66 | 132.08 (59.35-204.81) | 107.28 (60.13-154.44) | 103.43 (-12.54-219.4) | |
| SSL11 | 24 | 288.19 (145.45-430.93) | 247.93 (49.38-446.48) | 342.54 (46.26-638.81) | |
|  | 38 | 116.69 (50.27-183.11) | 136.09 (40.57-231.62) | 165.04 (-0.74-330.81) | |
|  | 45 | 98.11 (41.16-155.06) | 79.22 (-20.48-178.92) | 130.464 (12.33-248.6) | |
|  | 52 | 54.16 (19.72-88.6) | 51.28 (-8.79-111.35) | 89.21 (-30.08-208.51) | |
|  | 59 | 57.78 (11.48-104.08) | 92.21 (-69.72-254.15) | 40.68 (-24.65-106.01) | |
|  | 66 | 42.25 (11.26-73.24) | 39.13 (0.32-77.93) | 30.18 (-10.69-71.05) | |
| HlgB | 24 | 3956.53 (3160.72-4752.35) | 4137.39 (2963.67-5311.12) | 4534.39 (3312.97-5755.82) | |
|  | 38 | 2753.78 (2059.8-3447.76) | 2598.44 (1434.17-3762.71) | 2872.18 (1717.2-4027.16) | |
|  | 45 | 2260.23 (1576.54-2943.93) | 2191.81 (1083.56-3300.07) | 2514.82 (1501.39-3528.25) | |
|  | 52 | 1409.85 (963.25-1856.45) | 1371.47 (684.22-2058.72) | 1570.14 (919.38-2220.91) | |
|  | 59 | 946.32 (628.89-1263.74) | 992.89 (432.47-1553.32) | 1090.36 (592.58-1588.14) | |
|  | 66 | 753.59 (497.84-1009.34) | 732.69 (377.96-1087.42) | 823.07 (478.83-1167.31) | |
| LukD | 24 | 580.81 (485.95-675.67) | 620.46 (374.31-866.62) | 672.39 (381.85-962.94) | |
|  | 38 | 792.08 (651.64-932.51) | 775.78 (475.2-1076.37) | 919.5 (452.24-1386.77) | |
|  | 45 | 825.25 (534.57-1115.93) | 756.28 (553.26-959.3) | 795.18 (540.15-1050.21) | |
|  | 52 | 645.03 (509.87-780.19) | 642.22 (442.7-841.74) | 873.86 (427.66-1320.06) | |
|  | 59 | 702.08 (552.19-851.97) | 717.82 (497.81-937.83) | 747.07 (423.10-1071.04) | |
|  | 66 | 537.22 (463.22-611.22) | 582.88 (433.68-732.07) | 536.32 (322.41-750.23) | |
| LukE | 24 | 599.03 (502.63-695.43) | 631.5 (397.21-865.79) | 656.36 (423.63-889.09) | |
|  | 38 | 339.63 (276.13-403.12) | 336.72 (221.98-451.46) | 329.04 (166.88-491.19) | |
|  | 45 | 263.48 (208.05-318.92) | 299.34 (164.59-434.1) | 282 (126.87-437.13) | |
|  | 52 | 185.33 (151.93-218.73) | 187.91 (124.16-251.65) | 173.71 (93.72-253.71) | |
|  | 59 | 147.94 (124.92-170.95) | 149.32 (105.51-193.14) | 126.93 (94.11-159.75) | |
|  | 66 | 138.98 (115.49-162.48) | 112.06 (91.87-132.26) | 120.29 (103.16-137.41) | |
| LukF | 24 | 138.06 (88.12-188) | 145.86 (50.8-240.91) | 142.04 (48.94-235.14) | |
|  | 38 | 403.63 (225.03-582.22) | 310.22 (235.21-385.23) | 571.36 (424.11-718.61) | |
|  | 45 | 410.78 (297.7-523.86) | 284.94 (192.15-377.73) | 381.64 (252.01-511.28) | |
|  | 52 | 288.75 (195.58-381.92) | 264.78 (194.74-334.82) | 280.29 (159.19-401.38) | |
|  | 59 | 299.36 (191-407.72) | 255.54 (200.25- 310.82) | 344.64 (136.02-553.27) | |
|  | 66 | 221.86 (171.18-272.54) | 209.38 (169.78-248.97) | 310.54 (218.12-402.96) | |
| LukS | 24 | 866.33 (767.49-965.17) | 853.68 (684.78-1022.58) | 970.86 (778.62-1163.09) | |
|  | 38 | 564.67 (391.97-737.37) | 459.813 (335.54-584.08) | 467.57 (315.43-619.71) | |
|  | 45 | 421.36 (332.87-509.85) | 407.38 (264.69-550.06) | 459.57 (312.47-606.67) | |
|  | 52 | 268.61 (199.98-337.24) | 290.81 (214.65-366.97) | 245.57 (178.95-312.2) | |
|  | 59 | 178.84 (121.37-236.32) | 250.54 (108.2-392.88) | 169.29 (99.08-239.49) | |
|  | 66 | 187.48 (95.53-279.44) | 181.75 (106.48-257.02) | 129.68 (56.11-203.25) | |
| α-toxin | 24 | 4034.09 (3101.54-4966.65) | 3989.18 (2593.95-5384.41) | 4447.96 (2657.2-6238.73) | |
|  | 38 | 2757.88 (2112.94-3402.81) | 2506.31 (1598.93-3413.7) | 2915.75 (1223.48-4608.02) | |
|  | 45 | 2108.75 (1612.2-2605.3) | 2069.31 (1443.07-2695.56) | 2358.5 (1175.97-3541.04) | |
|  | 52 | 1525.28 (1155.45-1895.12) | 1469.34 (805.78-2132.91) | 1483 (706.86-2259.15) | |
|  | 59 | 1216.94 (913.26-1520.62) | 924 (648.65-1199.35) | 1166.46 (160.53-2172.4) | |
|  | 66 | 1312.81 (855-1770.63) | 1233.82 (537.87-1929.77) | 1186.36 (472.88-1899.83) | |
| SEA | 24 | 456.27 (213.36-699.17) | 355.14 (-25.21-735.5) | 598 (103.08-1092.92) | |
|  | 38 | 350.68 (260.13-441.24) | 280.59 (159.06-402.13) | 466.61 (277.73-655.48) | |
|  | 45 | 478.84 (318.57-639.12) | 266.03 (174.98-357.08) | 431.86 (282.25-581.46) | |
|  | 52 | 335.08 (214.56-455.6) | 171.19 (123.28-219.09) | 268.89 (177.69-360.1) | |
|  | 59 | 285.2 (163.98-406.42) | 202.54 (123.32-281.75) | 283.5 (173.51-393.49) | |
|  | 66 | 202.25 (122.33-282.17) | 160.41 (106.31-214.51) | 259.29 (166.92-351.65) | |
| SEC | 24 | 290.02 (76.93-503.1) | 238.32 (-87.19-563.84) | 67 (8.08-125.92) | |
|  | 38 | 171.33 (77.53-265.13) | 131.75 (78.97-184.53) | 111.46 (29.65-193.28) | |
|  | 45 | 214.13 (134.24-294.01) | 191.5 (76.39-306.61) | 135.36 (33.47-237.25) | |
|  | 52 | 151.59 (103.15-200.04) | 162.5 (70.42-254.58) | 143.54 (68.09-218.98) | |
|  | 59 | 152.48 (110.96-194.01) | 198.32 (41.36-355.28) | 162.32 (94.93-229.72) | |
|  | 66 | 144.7 (95.77-193.64) | 193.16 (69.93-316.38) | 144.43 (81.82-207.04) | |
| SED | 24 | 185.36 (132.31-238.41) | 184.61 (122.03-247.18) | 210.14 (103.68-316.6) | |
|  | 38 | 82.23 (58.12-106.35) | 75.5 (31.69-119.31) | 80.75 (5.34-156.16) | |
|  | 45 | 61.72 (37.42-86.02) | 59.5 (31.69-87.31) | 59.86 (10.36-109.36) | |
|  | 52 | 51.14 (36.55-65.73) | 42.59 (32.25-52.94) | 40.71 (13.71-67.72) | |
|  | 59 | 36.8 (25.93-47.66) | 30.32 (19.43-41.21) | 34.93 (14.23-55.63) | |
|  | 66 | 41.13 (32.04-50.21) | 37.75 (19.27-56.23) | 30 (4.93-55.08) | |
| SEG | 24 | 119.13 (49.34-188.91) | 141.82 (16.42-267.22) | 89.43 (52.2-126.66) | |
|  | 38 | 292.91 (226.46-359.35) | 281.44 (101.05-461.82) | 244.29 (117.91-370.67) | |
|  | 45 | 333.59 (256.31-410.88) | 376.41 (190.75-562.06) | 281.14 (146.04-416.24) | |
|  | 52 | 302.39 (229.27-375.51) | 413.66 (216.81-610.50) | 273.46 (201.53-345.4) | |
|  | 59 | 284.47 (226.69-342.25) | 346.96 (241.07-452.86) | 263.11 (128.73-397.48) | |
|  | 66 | 278.14 (210.69-345.59) | 284.75 (220.68-348.82) | 306 (157.06-454.94) | |
| SEJ | 24 | 441.98 (274.99-608.98) | 537.43 (282.24-792.61) | 387.75 (256.06-519.44) | |
|  | 38 | 530.19 (299.33-761.04) | 412.19 (153.26-671.11) | 512.96 (228.26-797.66) | |
|  | 45 | 384.22 (252.17-516.27) | 436.31 (139.28-733.34) | 529.25 (171.25-887.25) | |
|  | 52 | 259.05 (191.10-326.99) | 337.72 (194.89-480.55) | 302.57 (144.90-460.24) | |
|  | 59 | 207.75 (142.02-273.48) | 275.82 (116.79-434.85) | 298.61 (128.6-468.61) | |
|  | 66 | 165.45 (111.99-218.92) | 251.56 (143.38-359.74) | 215.18 (76.74-353.62) | |
| SEM | 24 | 310.17 (212.8-407.54) | 289.43 (158.53-420.33) | 334.18 (108.23-560.13) | |
|  | 38 | 452.11 (331.74-572.47) | 388.84 (254.35-523.34) | 589.82 (243.04-936.6) | |
|  | 45 | 516.59 (405.61-627.58) | 474.53 (416.46-532.6) | 556.86 (335.09-778.63) | |
|  | 52 | 464.39 (374.03-554.75) | 443.44 (309.97-576.91) | 532.89 (324.94-740.85) | |
|  | 59 | 443.2 (380.33-506.08) | 445.75 (326.96-564.54) | 440.39 (207.45-673.33) | |
|  | 66 | 340.72 (281.79-399.65) | 327.59 (244.31-410.88) | 316.64 (189.44-443.85) | |
| SEQ | 24 | 0 (0) | 0 (0) | 0 (0) | |
|  | 38 | 29.7 (7.48-51.92) | 47.22 (-4.32-98.76) | 47.88 (2.68-93.07) | |
|  | 45 | 50.47 (14.36-86.57) | 94.21 (7.87-180.6) | 44.43 (2.24-86.62) | |
|  | 52 | 58.38 (34.21-82.54) | 99.47 (23.69-175.24) | 73.11 (18.6-127.61) | |
|  | 59 | 82 (55.42-108.58) | 98.96 (27.86-170.07) | 68.86 (14.43-123.28) | |
|  | 66 | 87.83 (50.84-124.81) | 64.94 (33.75-96.12) | 59.21 (2.72-115.71) | |
| SER | 24 | 22.88 (-3.43-49.18) | 18.04 (-10.58-46.65) | 6.36 (-7.09-19.8) | |
|  | 38 | 111.11 (35.87-186.35) | 48.81 (-13.18-110.8) | 102.14 (-41.79-246.08) | |
|  | 45 | 104.3 (51.51-157.09) | 107.28 (35.8-178.76) | 98.64 (35.56-161.72) | |
|  | 52 | 101.94 (78.484-125.39) | 87.16 (39.13-135.19) | 59.43 (17.18-101.67) | |
|  | 59 | 86.58 (60.33-112.83) | 156.18 (-11.26-323.62) | 82.14 (58.33-105.95) | |
|  | 66 | 98.23 (74.68-121.79) | 106.5 (68.99-144.01) | 78.86 (48.39-109.32) | |
| TSST-1 | 24 | 143.27 (46.89-239.65) | 95.04 (25.19-164.88) | 116.39 (11.65-221.14) | |
|  | 38 | 454.18 (269.53-638.84) | 596.66 (193.09-1000.23) | 760.64 (106.5-1414.79) | |
|  | 45 | 836.3 (292-1380.59) | 885.64 (363.67-1407.62) | 645.89 (515.58-776.21) | |
|  | 52 | 765 (515.25-1014.76) | 750.09 (438.81-1061.38) | 519.54 (344.62-694.45) | |
|  | 59 | 673.32 (574.14-772.49) | 785.92 (400.61-1171.22) | 641.5 (367.6-915.4) | |
|  | 66 | 589.31 (486.81-691.81) | 660.14 (466.51-853.77) | 661.79 (438.09-885.48) | |
| ETA | 24 | 95.38 (15.51-175.24) | 48.43 (-22.84-119.69) | 87.36 (-23.83-198.54) | |
|  | 38 | 308.84 (216.89-400.8) | 213.34 (43.66-383.03) | 318 (65.6-570.4) | |
|  | 45 | 436.7 (309.38-564.03) | 273.88 (97.17-450.58) | 301.18 (192.9-409.46) | |
|  | 52 | 338.42 (199.38-477.47) | 265.31 (105.59-425.04) | 202.43 (109.56-295.3) | |
|  | 59 | 301.52 (192.71-410.32) | 287.79 (160.96-414.61) | 220.68 (158.84-282.52) | |
|  | 66 | 232.77 (178.48-287.05) | 299.06 (179.85-418.28) | 225.88 (127.48-324.27) | |
| ETB | 24 | 173.27 (100.56-245.97) | 204.18 (63.96-344.4) | 188.36 (29.68-347.03) | |
|  | 38 | 205.5 (107.21-303.79) | 145.38 (63.42-227.33) | 585.68 (-299.87-1471.22) | |
|  | 45 | 257.36 (174.13-340.59) | 219.97 (119.49-320.44) | 233.07 (115.33-350.82) | |
|  | 52 | 193.05 (145.13-240.96) | 243.03 (183.78-302.28) | 171.39 (121.66-221.13) | |
|  | 59 | 165.48 (133.59-197.38) | 195.46 (120.1-270.83) | 200.07 (37.09-363.05) | |
|  | 66 | 161.23 (133.26-189.21) | 137.5 (101.62-173.38) | 119.39 (66.99-171.79) | |
